# Performance of CMR stress/rest automated myocardial perfusion parametric mapping (perfusion map) as compared to manual plotting of the slope curves

**DOI:** 10.1186/1532-429X-16-S1-P13

**Published:** 2014-01-16

**Authors:** Ilan Gottlieb, Gabriel Camargo, Maria Eduarda Derenne, Leticia R Sabioni, Vania M Naue, Tamara Rothstein, Ralph Strecker, Andreas Greiser, Ronaldo S Lima

**Affiliations:** 1 Cardiac Imaging, National Institute of Cardiology, Rio de Janeiro, RJ, Brazil; 2 Cardiac Imaging, CDPI - Clinica de Diagnostico por Imagem, Rio de Janeiro, RJ, Brazil; 3 Cardiology, Federal University of Rio de Janeiro, Rio de Janeiro, Brazil; 4 Siemens AG Healthcare Sector, Erlangen, Germany; 5 Siemens LTDA, Sao Paulo, SP, Brazil

## Background

Cardiac magnetic resonance (CMR) stress/rest myocardial perfusion is usually assessed in clinical practice by qualitative visual analysis. There might be some added value in analyzing upslope perfusion curves, but those are labor and time consuming. We aimed to evaluate a new inline post processing tool that allows for completely automatic construction of upslope curves with parametric pixelwise construction of perfusion myocardial maps.

## Methods

We included 20 consecutive patients clinically referred for phamacological CMR myocardial stress perfusion with dypiridamol at target dose of 0.84 mcg/kg administered in 6 minutes - images were acquired 2 minutes after drug interruption. Patients were scanned using a 3T system (Magnetom Verio, Siemens, Erlangen) and images in stress first and 10 minutes later at rest were acquired during first passage of 0.1 mmol/kg of gadoteric acid meglumine (Dotarem, Guerbet, France). The sequence used for perfusion acquisition was a s-GRE with saturation pre-pulses before every acquisition. Matrix was 160 × 112, TI 90 to 110 ms, TPAT acceleration factor of 3 and 3 slices in total were acquired at the base, mid and apical regions of the left ventricle. Perfusion maps were built automatically by first applying a motion correction algorithm and then by building a map in which every pixel intensity corresponds to the upslope of myocardial signal increase during perfusion, without baseline correction. These slopes were also acquired by careful manual contouring of the myocardium in an offline workstation with dedicated software (CMR 42, Circle, Calgary). Analysis was performed blindly by segment in a total of 16 AHA segments per patient, carefully including most of the myocardium within a segment, but leaving small endocardial and epicardial margins in order to avoid partial volume averaging.

## Results

**Figure 1:**
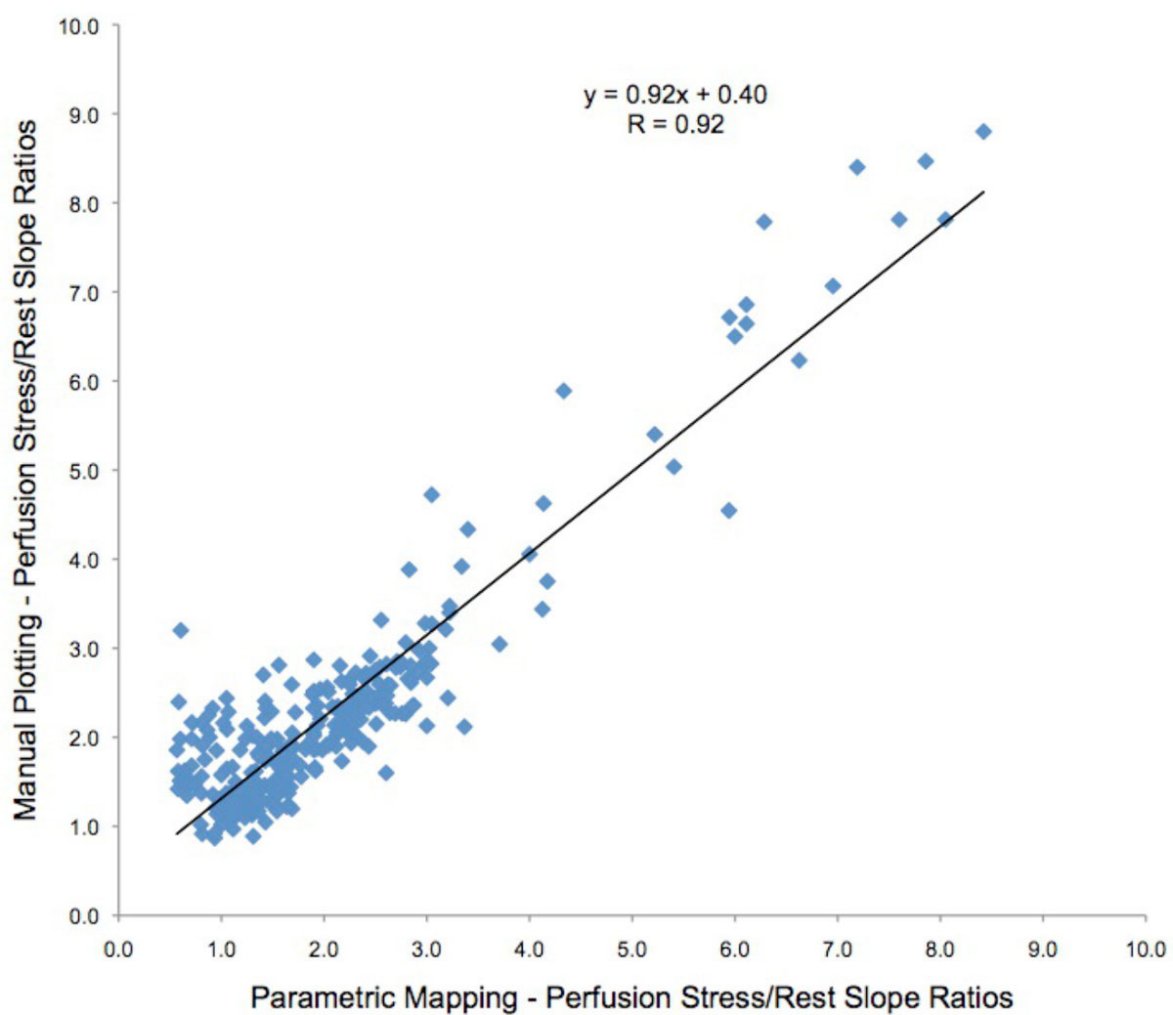
**Correlation between the automatic perfusion map and manual plotting of the upslope curves**.

A total of 320 myocardial segments were analyzed (no segment was excluded) from 20 patients (12 male), in which 6 (30%) had the clinical diagnosis of myocardial ischemia by an expert CMR reader. All patients completed the stress protocol uneventfully. Median pre-test probability of obstructive coronary disease was 11% (IQR 7-13%; range 1-94%). The Pearson's correlation coefficient R was 0.92, with a linear correlation formula y = 0.92x + 0.4, as seen in Figure 1. Apical segments had slightly lower correlation than mid and basal segments, respectively R = 0.85, R = 0.95 and 0.92.

## Conclusions

Automatic myocardial stress/rest perfusion mapping has excellent correlation with more labor and time intensive manual plotting of the curves, making it more suitable for clinical use.

## Funding

None.

**Figure 2:**
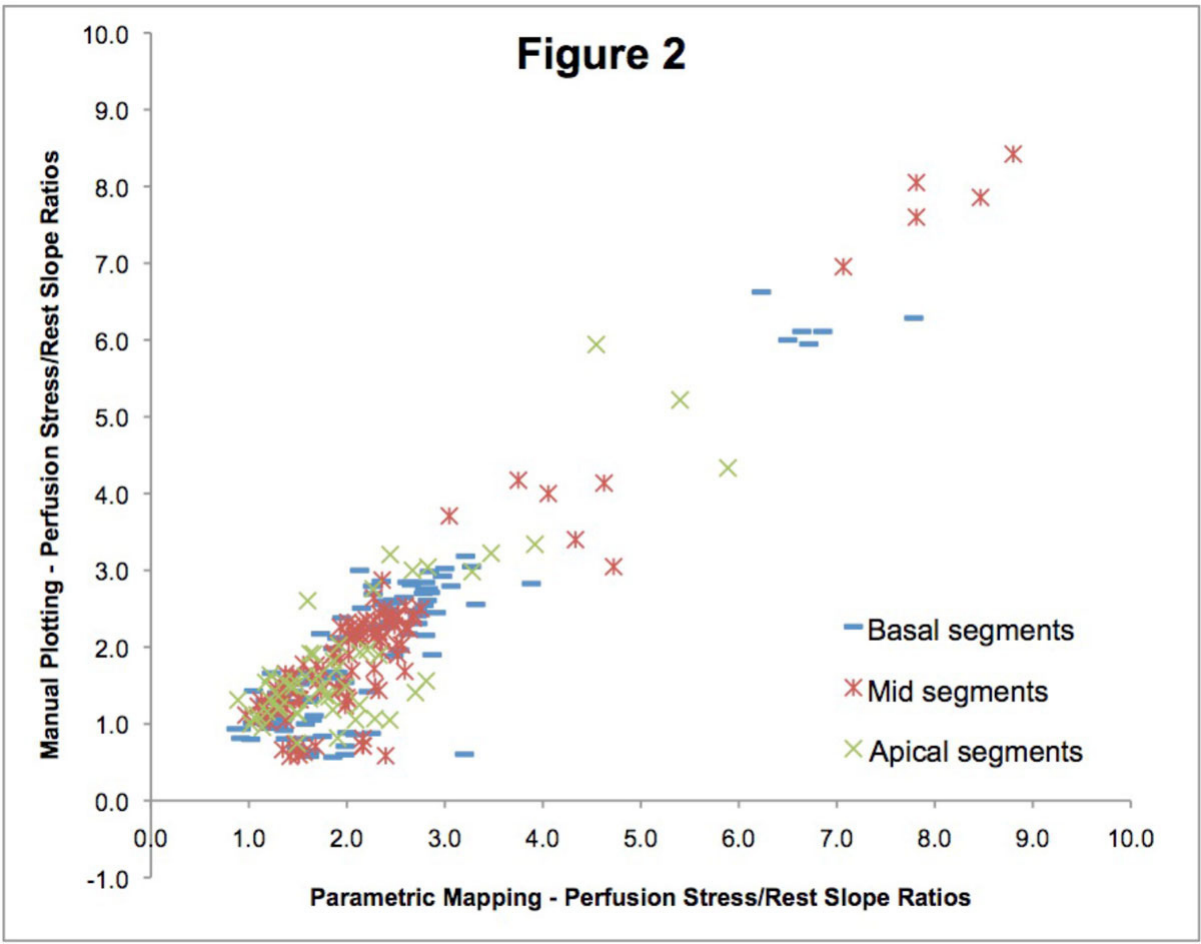
**Perfusion map compared with manual curve plotting divided by segment location in basal, mid or apical regions**.

